# Morgan County Medical Society

**Published:** 1866-12

**Authors:** 


### MORGAN COUNTY MEDICAL SOCIETY.

The Society met at the Court House, in Jacksonville, on Thursday, Nov. 8, 1866, at 11 o'clock A. M., Dr. R. E. McVey, the President, in the chair.

Dr. Warriner presented a specimen of castor oil, which had been deprived of the impurities of the commercial oil, but which had lost none of the valuable properties of this excellent remedy. He remarked that the commercial castor oil was often found adulterated with lard oil. He had succeeded in condensing, by chemicals, the lard oil, separating it from the castor oil.

Though his process was known only to himself, he offered to impart it to the Society if they deemed it necessary. The expressed opinion of the Society seemed to be that it was no breach of professional etiquette for him to keep his secret, and several members seemed to feel inclined to test the article.

Dr. Prince, having been called recently to Adams county as a witness in a suit for malpractice, described the injuries and made some general remarks suggested by the trial. The case was a rare dislocation—the forward dislocation of the tibia, slipping over the swell of the astragalus one inch forward of its natural position. The fibula was fractured, but the lower fragment clung to its natural attachments, and was not displaced. Several of the most eminent practitioners of the State, and of the West, were summoned to appear in the case—among others, Dr. Andrews, of Chicago. Not one had ever been called upon to treat, or had known before of, a dislocation of the kind. But little even can be found concerning it in any of the text books on surgery.

Dr. Prince remarked that a mere knowledge of anatomy did not at once interpret the case. It was a question whether the general practitioner should be better informed than the text books, or whether he should decline rare and obscure cases.

Dr. Edgar remarked that, as all physicians were liable to be called upon for surgery, they must take special pains in educating themselves for it; they would be liable for prosecution. More prosecutions arise from fractures and dislocations than from any other source in the practice of medicine. Educate yourselves specially to attend to fractures and dislocations. Never attend a case of fracture or dislocation unless you have an intelligent witness with you. He thought medical schools paid too little attention to the education of their students in these important particulars.

Dr. Prince thought that students should be taught to state "that in dislocations a cure cannot be guaranteed." He quoted Dr. Andrews, of Chicago, "who thought that medical men should not be held by the law responsible for bad practice, as they were not allowed by the law of the State to familiarize themselves with anatomy in the proper way."

Dr. Askew thought that prosecutions more often arose from the disagreement of doctors than from any other source. If the mantle of charity should be thrown over the little difficulties of practice, there would be much less trouble in the courts about doctors. Instead of trying to rise on their own merits, physicians too often attempt to build themselves a reputation on the failures or demerits of their neighbors. The State should be more rigid in its requirements of qualification, and should afford ample opportunity for the study of anatomy.

At 12½ o'clock adjourned to 2 o'clock P. M.

The first business in the afternoon was the reading of two quarterly reports of the Treasurer of the Society, which were accepted.

Dr. Prince not being prepared to give his promised essay, Dr. R. E. McVey, of Waverly, read an essay on "Phthisis and its relations with scrofula."

Dr. Prince asked the question, whether the science of medicine had progressed to that point that children, whose parents for generations had died of consumption, could be carried safely through to old age.

Dr. McVey thought he had, by the early use of cod liver oil, cured several cases of incipient phthisis.

Dr. Edgar thought that diseases of the liver were often mistaken for tuberculosis, and that many cases were possibly of this kind.

Dr. Henry Jones remarked that he had generally failed in his efforts to arrest the progress of this terrible disease, and considered it a very undesirable inheritance.

Dr. McVey was requested by the Society to furnish his interesting paper to some medical journal for publication.

The committee on "Fees" desired longer time in the preparation of their report, and Dr. Prince was added to it.

Society adjourned to meet at 11 o'clock A. M., on the second Thursday in December. C. T. WILBUR, M.D., *Secretary.*

---

*Hog Cholera in Ohio.*—It is a well known fact that the hog cholera is prevailing to a considerable extent throughout the country adjacent to the city. In view of this fact, and also that the price of pork is suffering a considerable decline from anticipated prices, we presume the temptation to avoid loss is too great for some men to withstand. We are informed by authority of undoubted reliability, that it is the practice of many pork owners to kill the hogs when first attacked by the disease, and send them for sale in our daily market.—*Cin. Gaz.*

